# Shape Representation of Word Was Automatically Activated in the Encoding Phase

**DOI:** 10.1371/journal.pone.0165534

**Published:** 2016-10-27

**Authors:** Tianyu Zeng, Liling Zheng, Lei Mo

**Affiliations:** Center for the Study of Applied Psychology, South China Normal University, Guangzhou, China; Hangzhou Normal University, CHINA

## Abstract

Theories of embodied language comprehension have proposed that language processing includes perception simulation and activation of sensorimotor representation. Previous studies have used a numerical priming paradigm to test the priming effect of semantic size, and the negative result showed that the sensorimotor representation has not been activated during the encoding phase. Considering that the size property is unstable, here we changed the target property to examine the priming effect of semantic shape using the same paradigm. The participants would see three different object names successively, and then they were asked to decide whether the shape of the second referent was more similar to the first one or the third one. In the eye-movement experiment, the encoding time showed a distance-priming effect, as the similarity of shapes between the first referent and the second referent increased, the encoding time of the second word gradually decreased. In the event-related potentials experiment, when the difference of shapes between the first referent and the second referent increased, the N400 amplitude became larger. These findiings suggested that the shape information of a word was activated during the encoding phase, providing supportive evidence for the embodied theory of language comprehension.

## Introduction

Language comprehension has always been the main focus in the field of cognitive research. How do people understand a word, a sentence or a paragraph? For nearly a decade, there have been two main theories about language comprehension: the semantic network theory and the embodied cognition theory.

The semantic network theory posits that the meaning of a concept is acquired through the connections with another relevant concept. Under this framework, when people process a specific concept, it will automatically activate other concepts closely related to it.

Recently, many researchers have begun to emphasize the activation of embodied experience during cognitive processing and have proposed the embodied cognition theory. The embodied approach to language comprehension claims that cognition is built on action, perception and emotional system of the brain [1–3]. Contrary to the semantic network theory, the embodied cognition theory proposes that a concept is acquired through the representation of sensorimotor experience [4]. For example, when we see the word “jackfruit”, we also “see” its color, “smell” its odor and “feel” its spine [5–7].

There are many studies that provided strong evidence for the embodied cognition theory. Glenberg and Kaschak (2002) have proved the action-compatibility effect (ACE), i.e., the certain action described in a sentence would affect the actual action of a participant [8]. Zwaan (2002) found that when the scenario described in the sentence matched the content in a picture, the reaction time would significantly decreased [9]. Other studies showed that a single word can also activate action representation. Rüschemeyer, Pfeiffer and Bekkering (2010) used object names in a lexical decision task; these objects were frequently used in a certain way, such as approaching the body (glass of water) or keeping away from body (key) [10]. The result showed that if the participants performed the same action as they typically performed with the object, their reaction would be faster than the opposite. This implied that the participants would extract the specific information of how the object was used during lexical retrieval, so the object name would prime the certain orientation of action and would influence the executing act. These findings have typically been obtained with object words and also with motion verbs [11–12].

However, some researchers have questioned the reliability of the evidence of embodied cognition. Mahon and Caramazza (2008) suggested that the sensorimotor representation effect observed in former studies could be explained by the abstract, non-embodied representation spreading into the system that was capable of sensorimotor representation [13]. Previous evidence could not prove that language comprehension processing must have included the activation of sensorimotor representation because the activation could be caused by the regulation from a higher level of the goal-driven process. This explanation also has experimental evidence. Louwerse and Jeuniaux (2010) found that when the task was related to special iconicity, the processing of a word was strongly affected by the spatial relationship of referents in reality [5]. However, when the task was to judge semantic relativity, the processing tended to be affected by the frequency of the word order. This result supports a goal-driven account of the sensorimotor activation of concept.

To examine this hypothesis, Hoedemaker and Gorden (2014) adopted Brysbaert’s (1995) numerical-priming paradigm to distinguish the encoding process from the goal-driven process [14–15]. Before Brysbaert, most of the studies about numerical comparison only asked participants to choose the bigger one or the smaller one among two numbers, but Brysbaert creatively proposed a new paradigm, in which participants were presented with the triplets of Arabic numerals and were asked to indicate whether the middle number was numerically in between the two outer numbers. In this paradigm, encoding of the middle number took place before a comparison decision must have been made, since all of the information needed to make a correct decision was only available after the third number had been processed. That was why the paradigm was capable of dividing the encoding phase and goal-directed phase in a single task.

In the past, a variety of experiments with regard to numerical magnitude have verified two main effects: one is the symbolic distance effect, in which, when choosing the larger one among two numbers, the reaction time would be shortened as the difference between the two numbers increased [16–18]. For example, choosing the larger number between 3 and 9 requires less time than making the same decision between 3 and 5. The other one is the numerical distance priming effect, in which the response to a numerical target is faster when it is preceded by a number that is close to it [15, 19, 20]. For example, processing the digit 5 takes less time when it was preceded by 3 than preceded by 1. These two adverse effects are also considered to reflect the different levels of processing: the numerical distance priming effect typically appears in the encoding phase, whereas symbolic distance effect occurs in the decision phase. In Brysbaert’s (1995) research, the result showed that the gaze duration time of middle number was shortened as the difference between the first number and middle number decreased, suggesting that the semantic information of the first number and middle number was activated during the encoding phase; also, after the third number showed up, the reaction time was shortened as the difference between numbers increased, showing the symbolic distance effect [15]. The result proved that the paradigm could effectively divide encoding phase and goal-directed phase in the same task.

Based on this, Hoedemaker and Gorden adopted the numerical priming paradigm to the word-priming paradigm by changing Arabic numerals to animal names; they wanted to examine whether the activation of the size property of a word occurs in the encoding phase or in the goal-driven phase [14]. The experimental task was to decide whether the size of the middle animal or the object was in between that of the two outer animals or the objects. The result showed that there was no distance priming effect in the encoding phase; when participants processed the first word and the middle word, the variety of size difference has no effect on the encoding time of the middle word. Additionally, the reaction time showed symbolic distance effect, that is, the larger the size difference between words, the quicker the participants reacted. Therefore, Hoedemaker concluded that the activation of the size property of animal names took place in the goal-driven phase. This finding supports the goal-driven account of Mahon and Louwerse, but not the embodied approach to language comprehension.

The work of Hoedemaker and Gorden (2014) can be regarded as a creative attempt to verify the traditional semantic network theory and the embodied cognition theory [14]. However, its result and conclusion might require further inspection. Generally speaking, the size property of a target is not important during the pattern cognition. Unlike color or shape, one’s representation of size tends to be more unstable and obscure. Former study has found that the mapping of the shape of referents would cause priming effect, that is “orange” can prime “ball”, “pizza” can prime “coin” due to their referents’ round shape [21–22]. However, according to Kang et al. (2011), the size of the referent has no effect on the lexical decision task [23]. Additionally, there are studies proposing that embodied effects only appear when perception information is the primary property [24], suggesting that the attribute of sensation itself may have influence on embodied effect.

Therefore, we believe that the result of Hoedemaker’s research could be caused by the characteristic of size property, and therefore is not strong enough to refute the embodied cognition. Because the perceptual representation of size is unstable, making the distance between word referents unclear and unnoticed, the size information of a word was not activated during the encoding phase. Here, we revised Hoedemaker and Gorden’s experiment design: rather than examining whether size perception was activated during the encoding phase of semantic processing, we adopted the same paradigm to investigate if shape perception was activated in the encoding phase, thus testing whether the sensorimotor representation of a word was activated automatically or was goal-directed. We assumed that when the shape of the first referent and the middle referent is similar, the encoding time of the middle referent should be shorter, showing a distance priming effect. This would represent that the shape attribute of the word was automatically activated in the encoding phase, and support the embodied language comprehension theory.

## Experiment 1

This experiment applied Brysbaert’s (1995) gaze-contingent triplet-comparison task to object name to examine whether the encoding time of the middle word would show a distance priming effect, thus investigating whether shape perception was activated during the encoding phase. The independent variable is the shape similarity between the first word and the middle word; the dependent variable is the gaze duration time of the middle word.

As Hoedemaker and Gorden (2014), we also examined the symbolic distance effect of the reaction time. The independent variable is the distance among the triplets, the dependent variable is the time that participants need to make a comparison decision.

This study was approved by the Research Ethics Committee of the South China Normal University and the methods were carried out in accordance with the approved guidelines.

### Materials & Methods

#### Participants

Twenty undergraduates (11 females) of the South China Normal University voluntarily participated in the experiment. Informed written consents were obtained from all of the participants. They were native speakers of Chinese, with normal or corrected-to-normal vision. All participants had not participated in material evaluation and did not know the goal of this research. The mean age of participants is 22.7, the standard deviation is 0.92.

Experimental stimuli came from two sources. First, we selected 30 commonly used object names from Holyoak (1979), Dean (2005) and Yee (2011) [25–27], and then we invited 10 undergraduates to brainstorm on these words and write down 3 objects that shared the same shape, had a similar shape, or had a different shape with the target object. We matched the 30 object names with these in pairs and invited 20 other undergraduates to rate the similarities of the shape on a scale of 1(very unlikely) to 7(almost the same shape). We eventually selected 25 words as the middle words and other words that have shape similarities with the middle word in three levels, including high similarity (average score above 5), medium similarity (between 2 to 5) and low similarity (below 2). To eliminate the disturbance from semantic correlation, we also asked another 10 undergraduates to rate the relativity between words in each pair. The result showed that most of the relativity scores between objects are approximately 1, indicating no relation. Any pairs of words with a score higher than 3 was deleted immediately.

The stimulus triplets were constructed as follows. Twenty-five object names served as targets appearing in the middle position of a triplet, whereas the other words served as flankers. Triplets were constructed so that when the semantic shape similarity between the first word and middle word was high, the similarity between the middle word and last word would be medium or low. When the similarity between the first word and the middle word was medium, the similarity between the middle word and the last word would be low or high. When the similarity between the first word and the middle word was low, the similarity between the middle word and the last word would be medium or high. Therefore, in one half of the trials, the similarities between the first word and middle word was higher than the one between the middle word and the last word (e.g., bulb-balloon-olive); in the other half of the trials, the similarities between the first word and the middle word was lower than the similarities between the middle word and the last word(e.g., shoes-balloon-bulb). Each of the middle words, repeated three times, comprised 75 trials. In addition, 25 object names were selected and constructed in the same way, comprising 75 filler trials and resulting in 150 trials per participant.

#### Procedure

Instructions and stimuli were presented using Experiment Builder. Eye movements were recorded from the participant’s right eye using an SR EyeLink 1000. The experiment stimuli are consisted of Chinese words, each word has two or three Chinese characters, the visual angle of each character is about 1.23 degree. At the beginning of each session, the tracker was calibrated using a 9-point procedure; calibration was checked between trials and the tracker was recalibrated when necessary. The participants sat in a well-lit room with a chin and a forehead rest minimizing their head movements. They were instructed to read the triplets silently and decide for each triplet whether the shape of the middle object was more similar to the left one (first object) or the right one (last object). The participants answered by pressing “f” or “j” on the keyboard. The experimenter monitored their eye-movements throughout the session.

**Fig 1:**
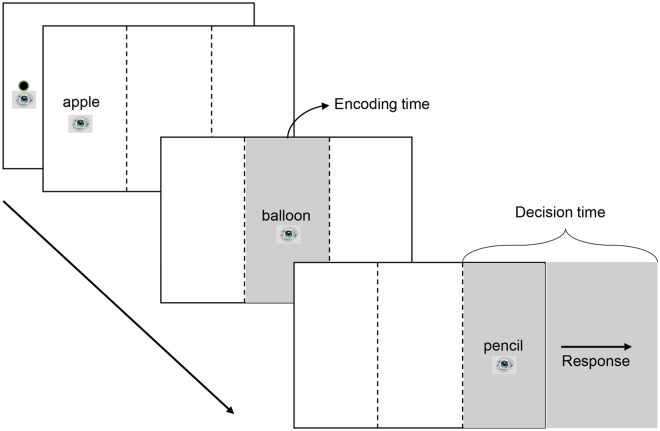
Presentation of stimuli and dependent measures in Experiment 1. Encoding time is measured as the gaze duration on the middle word. Decision time is the time from the onset of the last word until the indication of a response by a speeded key-press. A gaze-contingent display technique was used in which the words were masked except when the participant looked at them during the first reading pass from left to right. This eliminated preview and rereading of the first and middle word.

As illustrated in Fig 1, each trial started with a fixation point placed on the left side of the screen on the horizontal axis. Once this point was fixed, the next screen appeared, with the first word of the triplet slightly to the right of the fixed point. The middle and last word were in the center and on the right side of the screen on the same horizontal axis and remained unseen at first. Gaze-contingent invisible boundaries were placed approximately 120 pixels to the left of the middle and last word’s mask. Gaze contingencies were set up so that each word was visible only when the eyes entered the word’s region from left to right and was no longer visible after the eyes left its region to the right. This method of stimulus presentation prevented potential parafoveal preview or rereading of the first and middle word. When participants moved their gaze across the invisible boundary between the first and middle word, the middle word was unmasked and the first word was masked. The same event was repeated when the eyes crossed the invisible boundary between the middle and last word, so that the middle word was masked and the third word unmasked. Once the eyes left the first or middle word to the right, these items did not become visible again upon regressive eye-movement. The last word would remained visible until a response was made.

150 trials were presented randomly; participants could rest for 1 minute after each of 50 trials. Before the experiment started, participants need to complete 5 to 10 practice trials; they would received feedback on the accuracy and reaction time after each trial. However, no feedback would be given in the formal experiment.

The task provides two measures of interest: (a) Decision time is the interval between when the final object appeared on the screen and the execution of the manual response; it includes goal-based activation of task-relevant semantic properties because it covers the time during which all information required to perform the task was available. (b) Encoding time is the gaze duration on the middle word, and it is widely accepted in eye-tracking studies as a measure of encoding time for the item [28–30]. Following Hoedemaker (2014), gaze duration is the average of the sum of all first-pass fixation durations of a word [14].

There are lots of researchers that used eye movement index to investigate the cognition processing of reading. In most of the language comprehension studies, the gaze duration time was often used as referent of semantic coding. So, in the Experiment 1, encoding time is measured as the gaze duration on the middle word. Decision time is the time from the onset of the last word until the indication of a response by a speeded key-press. A gaze-contingent display technique was used, so that the words were masked except when the participant looked at them during the first reading pass from left to right. This eliminated preview and rereading of the first and middle word.

### Results

First, we analyzed whether the similarity between the first word and middle word would influence the gaze duration of the middle word.

**Fig 2:**
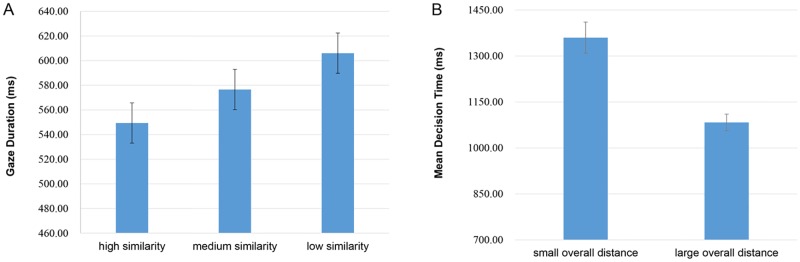
Gaze duration times and mean decision times result in Experiment 1. (A) Gaze duration times of middle word for trials with high, medium and low similarity in semantic shape between the first word and middle word, the result showed a distance priming effect. (B) Mean decision times for trials with small and large overall distance, the result showed a symbolic distance effect. Error bars indicate 95% confidence intervals.

Mean encoding times are shown in the Fig 2A. They showed a distance priming effect, with time decreasing as the similarity in semantic shape between the first and middle word increased. The gaze duration times were analyzed using repeated-measure ANOVA; there was a significant main effect of the shape similarity condition, *F*_*1*_(2,18) = 3.486, *p* = 0.041, *ŋ*_*p*_^*2*^ = 0.16; *F*_*2*_(2,23) = 3.613, *p* = 0.035, *ŋ*_*p*_^*2*^ = 0.13 (Mean encoding times: high similarity condition: 549.41±166.42ms, medium similarity condition: 577±212ms, low similarity condition: 606±197ms). There was no significant difference of encoding time between medium similarity condition and low similarity condition (*F*(1,19) = 2.13, *p* = 0.16, *ŋ*_*p*_^*2*^ = 0.10) or between medium similarity condition and high similarity condition (*F*(1,19) = 1.28, *p* = 0.27, *ŋ*_*p*_^*2*^ = 0.06). But, when the similarity of semantic shape is low, the encoding time of the middle word is significantly longer than when the similarity is high, *F*_*1*_(1,19) = 8.08, *p* = 0.01, *ŋ*_*p*_^*2*^ = 0.30; *F*_*2*_(1,24) = 7.823,*p* = 0.01, *ŋ*_*p*_^*2*^ = 0.25. The encoding time showed a distance priming effect.

Second, we analyzed whether the overall distance among triplets would affect the reaction time of participants.

In our research, the definition of distance is different from the former one. In Hoedemaker’s research, the distance between the first word and middle word equals the one between the middle word and last word, so they could use the difference of semantic size between the middle word and last word as an index of distance [14]. However, in our research, the similarity between the first word and middle word was not equal to the one between the middle word and last word, and as the difference between two similarities increased, it was easier for participants to make a comparison decision. Therefore, we defined a high similarity condition as 2, a medium similarity condition as 1, and a low similarity condition as 0, and calculated the relative similarity, respectively. When the difference between first similarity (similarity between the first word and middle word) and second similarity (similarity between the middle word and last word) equaled 2, it was called “large overall distance”; when the difference between first similarity and second similarity equaled 1, it was called “small overall distance”. After dividing all the trials into large overall distance and small overall distance, the average reaction times of the two conditions were calculated and analyzed.

As Fig 2B shows, participants reacted significantly slower when the overall distance was small, *F*_*1*_(1,19) = 15.90, *p* = 0.001, *ŋ*_*p*_^*2*^ = 0.46; *F*_*2*_(1,73) = 12.009, *p* = 0.00, *ŋ*_*p*_^*2*^ = 0.33 (Mean reaction times: small overall distance condition: 1360±455ms, large overall distance condition: 1083±243ms). The reaction time showed a strong symbolic distance effect.

The experiment result showed that the encoding time of the middle word was shortest when the first word and middle word had high semantic shape similarity; this distance priming effect indicated that the shape perception of object name was activated during the encoding phase. This result was contrary to Hoedemaker’s research, and proved our former hypothesis about size and shape property; that is, compared to shape, size information of a word is more unstable, making it inappropriate to be used in examining the distance priming effect. The decision time showed the symbolic distance effect; participants responded faster under large overall distance conditions, which is the same as Hoedemaker’s result.

To further confirm the behavior experiment results, we used a more sensitive method, the Event-related Potentials technique in Experiment 2, to examine the activation of perception representation during the language comprehension process.

## Experiment 2

This experiment used the more accurate and sensitive index, brain waveform, to verify the behavior result of Experiment 1.

In recent years, ERP technique was widely used in related research of semantic priming. Researchers have found that, compared to control condition, semantic-related target words would induce smaller N400 waveform. This semantic priming related N400 effect is very sensitive to semantic properties, and reflect the processing of semantic stimuli [31–34]. Also, researchers have found that, there was distinct N400 effect in the masked semantic priming condition, inferred that N400 represented the automatically activation process during word comprehension.

By using the Event-related Potentials (ERP) technique, we examined whether the shape perception was activated during the encoding phase or the goal-directed phase.

This study was approved by the Research Ethics Committee of the South China Normal University and the methods were carried out in accordance with the approved guidelines.

### Materials & Methods

#### Participants

Twenty-two undergraduates (14 females) of the South China Normal University voluntarily participated in the experiment and informed written consent was obtained from all participants. All participants were native speakers of Chinese, with normal or corrected-to-normal vision. They had not participated in the material evaluation or Experiment 1 and did not know the goals of this research. The mean age of participants is 21.6, the standard deviation is 1.89.

Experimental stimuli were constructed in the same way as in Experiment 1. To satisfy the minimum number of repetitions of an ERP experiment, we added 75 new triplets to the formal trials, including 225 trials in total. The addition of the trails was made in the same procedure in Experiment 1, including word brainstorming and rate of shape similarity.

#### Procedure

**Fig 3:**
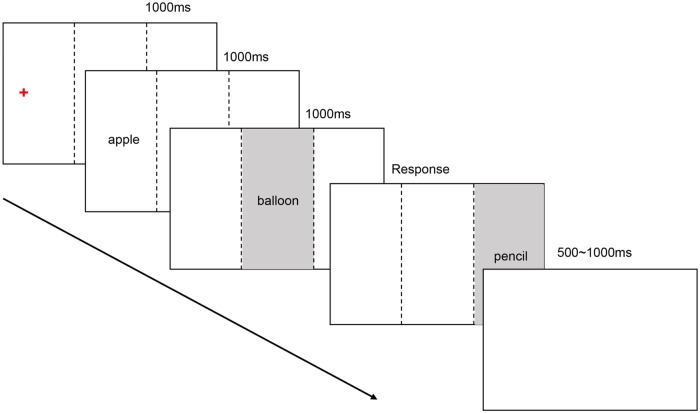
Presentation of stimuli in Experiment 2. Stimuli were presented subsequently in the same position and sequence like Experiment 1, with time interval of 1000ms. The first word and second word would appear on the screen for 1000ms, the last word would kept seen until the participant has made a reaction. Between each trial, there was a blank screen of 500ms to 1000ms randomly.

The experiment task was the same as Experiment 1, but instead of using gaze-contingent display technique, we used E-Prime 2.0 to control the time and place that stimuli presented. As illustrated in Fig 3, each trial started with a 1000ms fixation placed on the left side of the screen on the horizontal axis. Stimuli were presented subsequently in the same position and sequence like Experiment 1. The first word and second word appeared on the screen for 1000ms, and the last word remained visible until the participant had a reaction. Participants were asked to decide for each triplet whether the shape of the middle referent was more similar to the left one (first word) or the right one (last word). Between each trial, there was a blank screen of 500ms to 1000ms, randomly, and 225 trials were presented randomly as well. Participants could rest for 1 minute after each of the 40 trials. Before the experiment started, participants need to complete 5 to 10 practice trials.

#### ERP recordings

EEG activity was recorded continuously with scalp impedance constrained to be 5 kV or below throughout the recording session using Neuroscan SynAmps2 amplifier and 64 electrodes fitted on a Quick Cap based on the international 10–20 system. An electrode was placed on the participant’s left mastoid as the reference channel. Vertical EOG (VEOG) was recorded by 2 electrodes placed above and below the left eye, whereas horizontal EOG (HEOG) was recorded by two electrodes placed at the bilateral outer canthi of eyes. EEG Recordings were sampled at a rate of 1,000 Hz, filtered off-line using a 40Hz low-pass (zero-phase) and segmented into 1050ms epochs comprised of a 100ms pre-stimulus baseline and a 950ms post-stimulus interval. Segments containing artifacts exceeding ±100 mV were rejected before averaging. In each condition, mean amplitude ERPs for target words was computed.

### Results

ERP data analyses was conducted on electrophysiological waveforms evoked by object naming in all effective trials after averaging three proximal electrodes to isolate six regions of interest (ROIs) along the sagittal and the coronal cerebral axes according to previous studies: left-anterior (F5, F7, FC5), mid-anterior (Fz, FCz, Cz), right-anterior (F6, F8, FC6), left-posterior (P5, P7, CP5), mid-posterior (CPz, Pz, POz), and right-posterior (P6, P8, CP6). Refer to the previous research, we analyzed ERP data for the two experimental conditions in two time windows, 400–500 ms after the middle word (look for N400 index to examine the priming effect between the first word and the middle word) and 400–700 ms after the last word (look for LPP index to examine the decision process of different overall distance conditions). A 2×6 repeated measures ANOVA with shape similarity and ROI location as within-subject factors was conducted on the data pertaining to each of the two time windows respectively. Degrees of freedom were adjusted using Greenhouse-Geisser correction to account for data non-sphericity.

As in Experiment 1, we divided the trials into large overall distance and small overall distance group, and calculated the reaction time of each condition. The result showed that participants reacted significantly slower when the overall distance was small, *F*(1,21) = 16.42, *p* = 0.001, *ŋ*_*p*_^*2*^ = 0.44 (Mean reaction times: small overall distance condition: 1337±701ms, large overall distance condition: 1063±509ms). The reaction time showed a strong symbolic distance effect.

**Fig 4:**
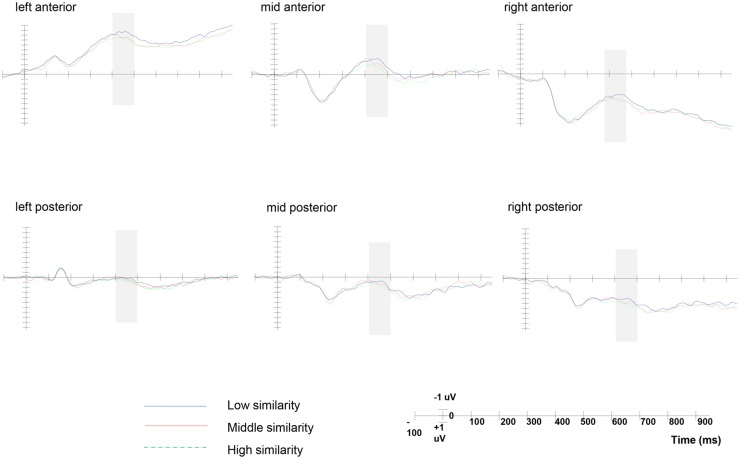
Grand average ERPs at six ROIs in Experiment 2 (400–500ms). The origin of the time-axis (0ms) is placed at the onset of the target word, ERPs elicited in the low similarity condition (dotted line) were significantly more negative than the high similarity condition (solid line) in the 400- to 500-ms interval after middle word onset across six ROIs (light grey shading), indicating the shape attribute of the word was activated during encoding.

The result of the ERP data of 400–500ms time window (Fig 4) showed a marginal significant main effect of the shape similarity condition, *F*(2,42) = 3.452, *p* = 0.056, *ŋ*_*p*_^*2*^ = 0.141 (Mean amplitude: high similarity condition: 0.71±0.36μV, medium similarity condition: 0.38±0.46μV, low similarity condition: -0.12±0.33μV), the waveform became more negative as the similarity of shape decreased. There was also a significant main effect of ROI location, *F*(5,105) = 80.137, *p* = 0.000, *ŋ*_*p*_^*2*^ = 0.792; the interaction between shape similarity and ROI location was not significant, *F*(10,210) = 0.816, *p*>0.05.

**Fig 5:**
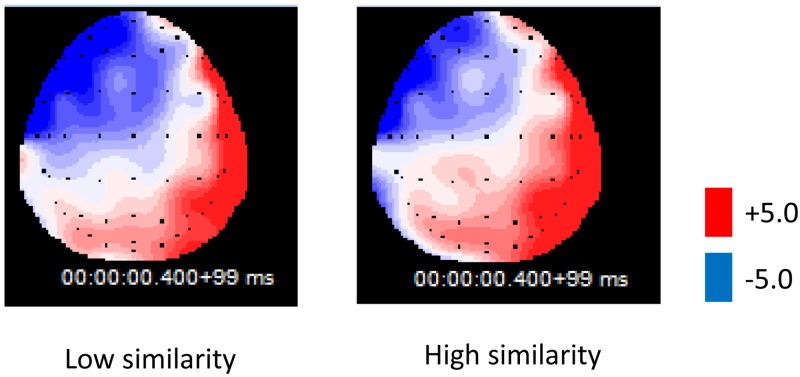
Topography of ERPs for the low similarity and high similarity condition (400–500ms).

Pair-wise contrast result showed that there was significant difference between high similarity condition and low similarity condition, *F*(1,21) = 16.04, *p* = 0.001, *ŋ*_*p*_^*2*^ = 0.43, with more negative ERPs for the low similarity condition than the high similarity condition. The topography of N400 for the low similarity and high similarity conditions showed that the left anterior area has more negative waveform than the others (Fig 5). In accordance with previous research, that the encoding of semantic knowledge mainly relies on the left frontal lobe and left temporal lobe.

The experiment results showed ERPs evidence of the semantic priming effect: in the 400- to 500-ms interval after middle word onset, the waveform of the low similarity condition was more negative than the high similarity condition. This result is in accordance with Kellenbach’s (2000) research, which proved that if the shape perception of object name was activated during the encoding phase, the former word could prime the processing of the word with similar shape [34]. When the shape of former object was different from the target object, the participant would notice the incongruity between two objects, thus resulting in a larger N400 waveform. In general, the result is in accordance with the Experiment 1, providing evidence for the activation of sensorimotor representation during language comprehension.

**Fig 6:**
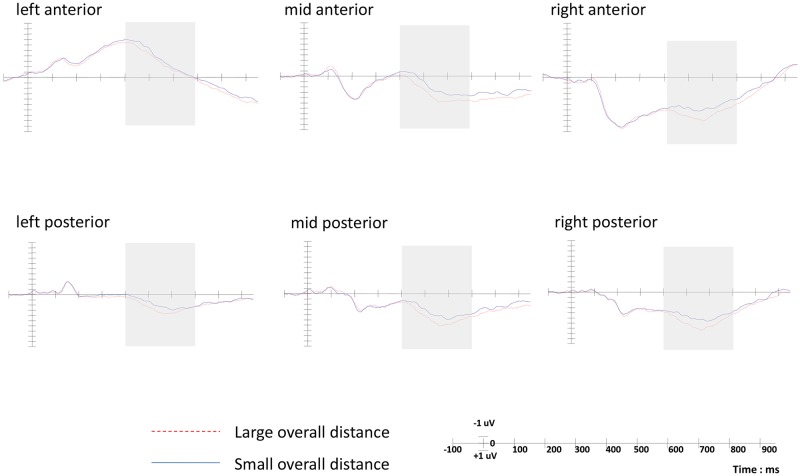
Grand average ERPs at six ROIs in Experiment 2 (400–700ms). The origin of the time-axis (0ms) is placed at the onset of the target word, ERPs elicited in the large overall distance condition (dotted line) were significantly more positive than the small overall distance condition (solid line) in the 400- to 700-ms interval after third word onset across six ROIs (light grey shading).

**Fig 7:**
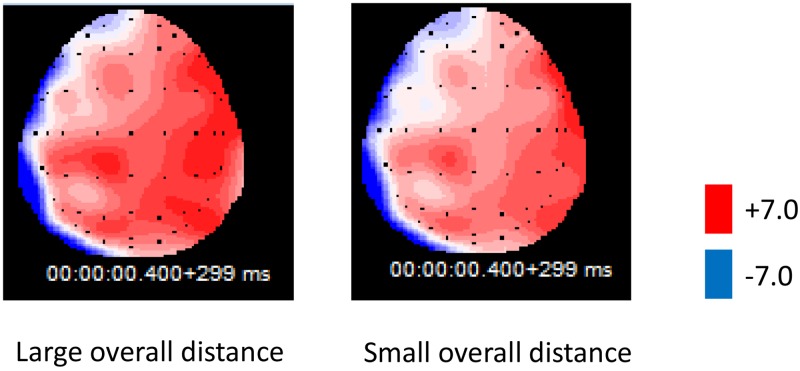
Topography of ERPs for the large overall distance and small overall distance condition (400–700ms).

The ERP data of 400–700ms time window (Fig 6) showed significant interaction between overall distance and ROI location (*F*(5,105) = 2.85, *p* = 0.04, *ŋ*_*p*_^*2*^ = 0.12). There was a significant main effect of the overall distance condition (*F*(1,21) = 5.65, *p* = 0.027, *ŋ*_*p*_^*2*^ = 0.21) and ROI location (*F*(5,105) = 34.83, *p* = 0.000, *ŋ*_*p*_^*2*^ = 0.62). To more specifically characterize the spatial distribution, post-hoc paired *t* tests were conducted to compare the 400–700ms component for the two conditions in each ROI. We found more positive ERPs for the small overall distance condition than the large overall distance condition, which was significant in the mid region and right region, whereas there was no significant difference between the two conditions in the left region. From the topography of LPP (Fig 7), we can clearly see that the LPP was distributed more broadly on the right hemisphere, which is consistent with previous findings [35–36].

In the 400- to 700-ms interval after last word onset, the waveform of the large overall distance condition was more positive than the small overall distance condition. Former studies have proposed that the late positive potentials can reflect the function regulation of attention resource, in the research of picture complexity and emotion arousal, Bradley (2007) found that compared to complicated picture that require more attention resource, the LPP waveform was more positive when participants processed simple picture [37]. Our research result was consistent with Bradley’s finding; with large overall distance, it is easier for participants to make a judgment, thus inducing a larger LPP waveform.

## Discussion

Traditional semantic network theory posits that knowledge derives from abstract propositional symbols and constructed semantic networks according to certain rules. The concept was seen as an abstract symbol, with no connection to one’s sensorimotor experience. Recently, supporters of the embodied cognition theory have conducted a series of studies proposing that the embodied representation of concepts was activated automatically. The embodied cognition theory posits that the meaning of a word is rooted in the perception and motor system, thus one would activate the related sensorimotor experience from the past while extracting the meaning of a certain word. However, objectors claim that the research evidence of embodied language comprehension is not strong enough to prove that the sensorimotor representation was activated automatically, and it could be affected by the goal-driven process instead. That is to say, the evidence of priming effects in the embodied semantic theory can be explained by the semantic symbol theory as well because, in these studies, if participants only activated relevant sensorimotor representation until they made a comparison decision, it would also generate the priming effect. Therefore, we can only prove that the priming effect stands for embodied cognition theory if we can make sure that the sensorimotor representation was activated during the encoding phase.

To solve the dispute, Hoedemaker (2014) has adopted the numerical priming paradigm of Brysbaert (1995) and distinguished the encoding phase from the goal-driven process to examine at which level the activation of word perception occurred [14–15]. The result showed that there was no distance priming effect in the encoding phase; the distance of size between word referents had no influence on the encoding time, which means that the size attribute was not activated during the encoding phase. This result supported the goal-driven theory and opposed the embodied view of language comprehension.

However, when we adopted the same paradigm as Hoedemaker and has only changed the target property from size to shape, the result has changed as well. In our research, the result showed distance-priming effects in the encoding phase, indicating that the shape property was activated; during the comparison task, it showed symbolic distance effects, which reflected goal-driven process. Our research proved that the shape property was activated in the encoding phase, providing evidence for the automatic activation of sensorimotor representation of embodied language comprehension. In Experiment 1, we chose the encoding time and decision time as the experiment indices. And by using the gaze-contingent display technique, we assured that the participant could only encode the word during the first-pass of reading from left to right, effectively eliminating preview and rereading effect that might have reduced the value of viewing time as a measure of encoding. The result showed that as the shape similarity between the first word and middle word increased, the encoding time of the middle word decreased relatively, which was contrary to the result of Hoedemaker’s study. For the comparison task time, as the overall distance increased, the reaction time decreased gradually in accordance with previous research. In Experiment 2, we used event-related potentials technique to examine the shape-priming effect between the first word and middle word. The result showed that when the shape of the first word was different from the middle word, participants would generate more negative N400, suggesting that they’ve noticed the incongruity of the shape of the word referent, which also implied that the shape property of the middle word was activated during the encoding phase.

Connell’s (2010) research has found that even when the specific conception attribute was being told before the task, it still took more time for participants to process touch-related words (like warm, itchy) than other words about vision, audition, gustation and olfaction [38]. The tactile disadvantage in perception process suggested that target attribute also has influence on cognition process. Thus, it is necessary for us to revise the experiment conducted by Hoedemaker by changing the target property from size to shape. We believe that the absence of distance-priming effects in Hoedemaker’s research could be caused by the inappropriate choice of semantic property. As we have mentioned before, the object’s size property is unimportant during pattern recognition, and the size representation of an object tends to be less stable and accurate than shape and color. Size itself is a relative concept, for example, cat is quite huge to the ant, but it is only a small fuzzy ball comparing to an elephant, and it is difficult for us to say whether the size difference between cat and ant equals to the one between elephant and cat. Kang et al. (2011) has discovered that in the lexical decision task, the word with a referent of larger size does not have processing advantage and has no difference from a word with a referent of smaller size [23].

However, there are many studies that have proved the priming effect of shape property. For example, it has been proved that words sharing the same shape can facilitate processing: “orange” can prime “ball”, “pizza” can prime “coin”, etc. [21–22]. An eye movement study using visual word paradigm has discovered that compared to irrelevant target, people are more likely to stare at the object that has the same shape as the prime word [39]. Kellenbach, Wijer and Mulder (2000) discovered that in a lexical decision task, if a prime word that shares similar shape with the target word was presented, the waveform of N400 would be smaller (like “button” prime “coin”) [34]. In their research, Rommers, Meyer and Huettig (2013) adopted a look-and-listen task in which participants were asked to listen to the sentences carefully and told that they were free to look at whatever they wanted to [40]. They first asked participants to listen to a sentence, such as “In 1969, Armstrong became the first man to step on the moon”, and 500ms before the target word “moon” came up, there would be four pictures on the screen. The result showed that even before hearing the shape-related target word (“moon”), participants were more likely to look at the object picture that has same shape of the target word referent (like “tomato”). Obviously, the shape information of a word was still activated even without explicit cue or shape-related tasks. In Stanfield’s (2001) study, participants would first read sentences with certain directions, such as “John has put the pencil into the pencil vase / drawer”, and then, when presented with object pictures with directions (vertical / horizontal), the participant was asked to judge whether the object had appeared in the preceding sentence [41]. The result showed that the processing speed was faster when the direction of the sentence matched the object [41–42]. Similarly, after reading “The soldier saw an eagle flying in the sky”, participants would respond faster when they saw a picture of an eagle stretching its wings than one with wings folded. To sum up, previous research has indicated that shape attribute representation is far more stable and distinct than size attribute and is more likely to be activated. Thus, our research chose shape attribute of the word’s referent as the target property for good reasons.

In addition, although in our research, the experiment task has direct relation to the shape property, the paradigm we used effectively assure that participants can only make decision after the third word was processed. To avoid participants’ comparing the similarity of shape before the third word showed up, we’ve added 75 filler trials besides 75 formal trials. In the filler trials, the shape similarity between three words could be very high or very low, making it really difficult for participants to make decision based on the first two words. Also, the reaction time showed that the mean gaze duration of second word was around 600ms, but the mean reaction time of third word was between 1100ms to 1400ms, distinctly longer than the second’s. This result suggested that the reaction time of the third word included the encoding time and the time to compare the shape similarity.

We also adopted ERP technique in Experiment 2 to further verify the former behavior result. In the past, a large amount of language studies have proven that the N400 index reflects the semantic processing of word and is one of the research indices of early cognition process. Thus, the more negative N400 in low similarity condition strongly demonstrated that the shape representation was activated in the encoding phase.

In conclusion, our research revised Hoedemaker’s numerical priming paradigm and chose shape attribute of a word’s referent as the target property. The result showed that the encoding time of the middle word has a distance priming effect, proving that the shape property of a word’s referent was activated during the encoding phase, which in turn provides evidence for the embodied view of language comprehension. In this research, we adopted the same paradigm as Hoedemaker and changed the target property from size to shape, but the experiment result turned out differently. On one side, this suggested that certain attribute has effect on the cognitive process; on the other side, it is undeniable that, to some extent, this may also showed that the embodied cognition theory is still unstable today. Our research result has offered experiment evidence for the embodied theory, but whether it can completely prove the embodied cognition theory of language still need further verification in the future.

## Supporting Information

S1 File Experiment data of experiment 1. (ZIP)

S2 File Experiment data of experiment 2. (ZIP)
